# Glucocorticoid Signaling-Associated Gene Expression in the Hippocampus and Frontal Cortex of Chronically Isolated Normotensive and Hypertensive Rats and the Responsiveness to Acute Restraint Stress

**DOI:** 10.3390/ijms262412050

**Published:** 2025-12-15

**Authors:** Alexey Kvichansky, Liya Tretyakova, Yulia Moiseeva, Viktoriia Ovchinnikova, Diana Mamedova, Olga Nedogreeva, Natalia Lazareva, Natalia Gulyaeva, Mikhail Stepanichev

**Affiliations:** Institute of Higher Nervous Activity and Neurophysiology, Russian Academy of Sciences, 117485 Moscow, Russia; al.kvichans@ihna.ru (A.K.); kind.dr.lee@gmail.com (L.T.); julia_moiseeva@mail.ru (Y.M.); ovchinnikovavic@gmail.com (V.O.); mamedova.diana2@yandex.ru (D.M.); nedogreewaolga@gmail.com (O.N.); nalaza@rambler.ru (N.L.); nata_gul@ihna.ru (N.G.)

**Keywords:** social isolation, hypothalamus–pituitary–adrenal system, sympatho-adrenal system, frontal cortex, hippocampus, corticosterone, gene expression

## Abstract

Genotypic characteristics may determine the body’s response to stressful conditions as well as its susceptibility to cardiovascular diseases and stroke. Old age worsens the course of these diseases, and often concomitant hypertension can negatively affect brain function, especially in cases of social isolation. In this work, we studied how social isolation and hypertension affect the transcription activity of genes associated with glucocorticoid signaling in the rat brain. The study was performed on 10-month-old rats of the outbred Wistar stock (*n* = 48) and the inbred spontaneously hypertensive (SHR) strain (*n* = 28). The animals of each genotype were divided into groups, one of which was kept in home cages in groups of 3–4 individuals, and the other in single cages for 3 months. Physiological parameters and plasma corticosterone were controlled before the start and after 3 months of isolation. Each group was additionally divided into two subgroups: one subjected to 1 h of restraint stress, and changes in blood glucose and corticosterone levels were assessed. At the end, the levels of *Nr3c1*, *Nr3c2*, *Hsd11b1*, and *Fkbp5* mRNAs were measured in the hippocampus and frontal cortex using the Q-PCR technique. After isolation, weight gain stopped in SHRs, although blood pressure did not change, and heart rate increased in rats of both genotypes. In response to restraint, there was practically no increase in corticosterone in isolated Wistar rats, whereas in SHRs, there were significant glucose and corticosterone responses. Significant disruptions in the system responsible for corticosterone-activated signaling cascades were found in the brains of SHR rats. The transcriptional activity of genes encoding corticosterone receptors and proteins regulating their action was reduced in the hippocampus and frontal cortex in SHRs compared to Wistar rats. However, neither isolation nor acute stress significantly affected the contents of transcripts studied. Meanwhile, after isolation, the relationships between the expression of these genes changed significantly, in different directions, in rats of the studied genotypes, both within and between brain structures. Thus, the SHR genotype is associated with persistent changes in the brain that affect the expression of glucocorticoid-associated genes. This indicates a more complex regulation of the stress response, not limited only by the feedback system within the hypothalamic–pituitary–adrenocortical or sympatho-adrenomedullary systems, but operated at the level of the limbic system and the cerebral cortex.

## 1. Introduction

The hypothalamus–pituitary–adrenocortical (HPA) and sympatho-adrenomedullary systems (SAS) are two main players in the neuroendocrine orchestration of the body’s response to stress [1,2,3]. Physical stressors activate structures of the brainstem, including noradrenergic neurons of the locus coeruleus, which send their projections to almost all brain regions and activate them. They also activate a neuroendocrine response of the HPA system to various stressors. Psychological stressors such as disruption of social interactions not only stimulate the secretion of pituitary corticotrophs but also should be processed in the frontal cortex and hippocampus [4,5,6].

Social isolation is a form of impairment of social relationships that has become a subject of many studies due to its popularity as a form of prevention against COVID-19. However, meta-analysis of literature data performed a long time before the pandemic demonstrated that both physical and subjectively perceived social isolation or loneliness equally shortened life expectancy [7]. Many authors of analytical reviews reported aging as one of the main factors that influence isolation consequences, although current data are very variable and heterogeneous [7,8,9].

Genotype may play a significant role in the body’s response to stress from yeast to humans [10,11,12]. Genotype-predetermined individual differences govern metabolic maintenance, including energy costs [11], as well as structural modifications associated with various stages of adaptation to stress [13]. These processes depend on complex gene × gene and gene × environment interactions. Primarily, genes encoding proteins involved in the functioning of the sympathetic nervous system and the HPA system affect variations in the stress response [12]. Animals of different strains provide a unique opportunity to study the effects of genetic background on structural and functional alterations evoked by chronic or acute exposure to stressors. Spontaneously Hypertensive Rats (SHRs) were bred from Wistar rat stock based on spontaneous hypertension. Increased sympathetic activity is a feature of SHRs, which is manifested long before the manifestation of hypertension [14,15]. In SHRs, sympathetic hyperactivity and parasympathetic insufficiency may underlie the efficient adaptation of the cardiovascular system to repeated restraint stress [16], supporting data from human studies mentioned above.

Despite numerous studies conducted in humans, data on alterations of the indices of activity of the HPA system studied in blood after social isolation of various durations are contradictory. Some researchers report a positive correlation between the levels of loneliness and plasma cortisol [17,18], while others report a negative correlation between these indices [19]. Similar inconsistencies can be seen in rodent studies. Thus, 6- [20] or 13-week [21] social isolation of adolescent rats led to an increase in blood corticosterone (CORT), in the latter study, only in females. A similar effect was observed in adult male rats after 5 weeks of social isolation [22] and in 1.5-year-old male rats after 12 weeks of isolation [23]. Sánchez et al. [24] demonstrated that individual housing of rats for 8 weeks starting from day 16 after partition resulted in a decrease in CORT blood content. Many authors did not find any changes in circulatory CORT levels after social isolation of various durations applied to juvenile [25,26,27,28,29,30,31,32] or adult [33,34] male or female rats of different strains.

Social isolation can modify the body’s response to acute stress. For example, short-term crowding or its combination with restraint after social isolation increased the level of CORT in plasma to some extent, but this effect was less expressed in isolated compared to group-housed animals [30]. It seems that the pattern of the response probably depends on the severity or origin of a stressor. Thus, isolated rats responded with higher CORT to restraint but not cold exposure [35]. In this case, the CORT increase was again lower compared to control rats. Taken together, these data show impaired functioning of the HPA system in rats subjected to social isolation.

Population studies show that long-term loneliness is associated with elevated arterial pressure in humans independently on age, sex, race, or severity of stress during the isolation period [36]. Under laboratory conditions, the subjective sense of loneliness positively correlated with diastolic arterial pressure and awakening cortisol levels in women [37]. Social isolation results in functional impairments of the cardiovascular system in animals as well. Juvenile male and female mice subjected to isolation for 2 months exhibited an elevated heart rate, and only females had increased arterial pressure [38]. Despite this, in SHRs, social isolation for 7 months after 21 days of age led to a decrease in arterial pressure, which was not observed in normotensive animals [39]. Short-term 5-day social isolation of rats resulted in an increase in systolic arterial pressure and tachycardia, which were prevented by daily 1 h handling or return to group housing [40]. It was hypothesized that dysfunction of the HPA system and SAS causes these impairments [40,41]. Interestingly, isolated housing before social territorial stress led to increased systolic arterial pressure in normotensive Wistar–Kyoto (WKY) rats, whereas the CORT content in the blood was decreased [42].

The aim of the present study was to tackle several questions. Did essential hypertension modify the capability of the body to adapt to long-term social isolation on the level of physiological parameters and circulating CORT or not? How did it contribute to the response of the body to acute heterotypic stress, such as restraint? How were these modifications reflected in the changes in transcriptional activity of the genes associated with CORT signaling in the frontal cortex and hippocampus, two brain regions involved in the central control of a stress response? For this purpose, we used SHRs as a model of essential hypertension and Wistar rats as a normotensive control. We studied mRNA expression of four genes related to CORT signaling in the brain: specifically, the *Nr3c1* and *Nr3c2* genes encoding glucocorticoid and mineralocorticoid receptors, respectively; the *Hsd11b1* gene encoding 11β-hydroxysteroid dehydrogenase type 1, an enzyme converting inactive into active glucocorticoids in the brain; and the *Fkbp5* gene encoding the FK-506-binding protein 51 (FKBP5/FKBP51), modulating the activity of glucocorticoid receptors and, thus, regulating the effects of CORT.

## 2. Results

### 2.1. Changes in Body Weight and Arterial Pressure in Rats

**Figure 1:**
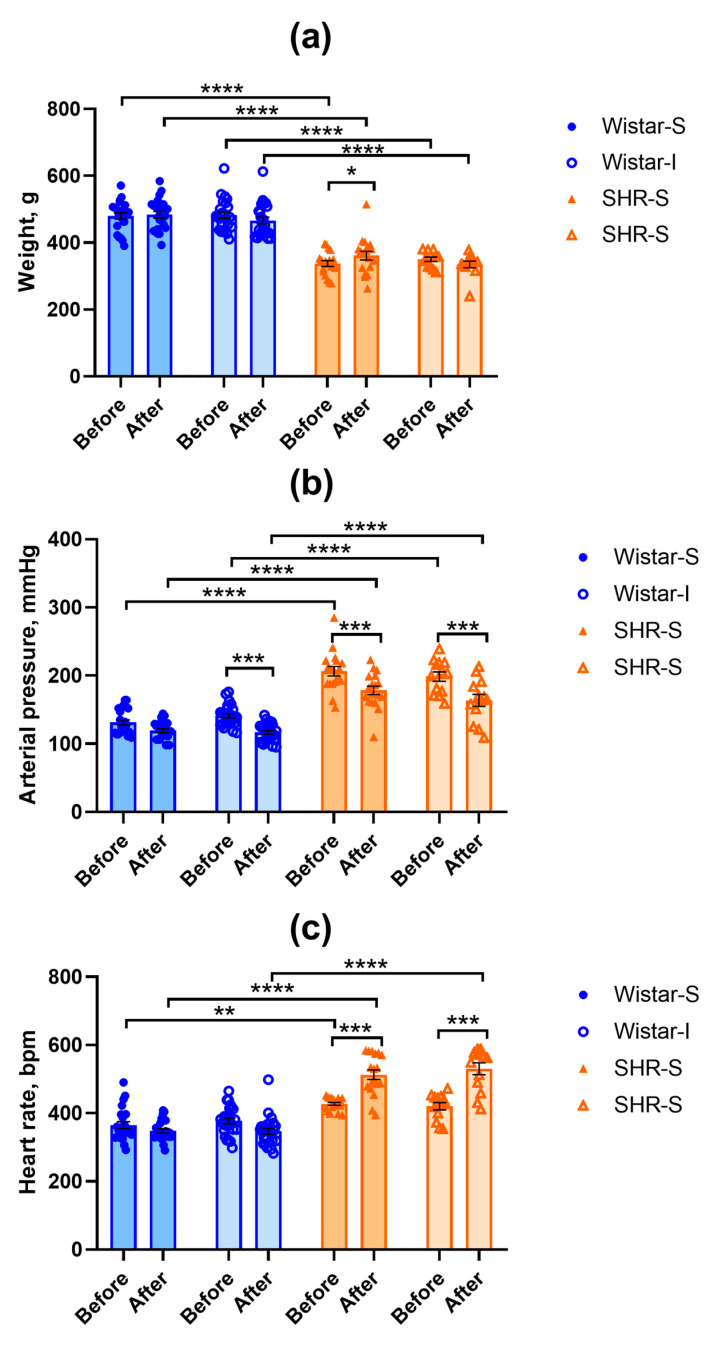
Effects of social isolation on the body weight (**a**), systolic arterial pressure (**b**), and heart rate (**c**) in Wistar rats and SHRs. All the measurements were performed before the start of the experiment and after 12 weeks of isolation (points Before and After on abscissa axis, respectively). Wistar-S and SHR-S are the groups of rats housed in group conditions, and Wistar-I and SHR-I are the groups of rats housed in isolated conditions. The differences between the groups were calculated using repeated-measures ANOVA followed by Tukey test for unequal N for multiple comparisons. The differences are significant at *, *p* < 0.05, **, *p* < 0.01, ***, *p* < 0.001, and ****, *p* < 0.0001 according to the post hoc Tuley test for unequal N. Data are presented as M ± s.e.m.

Data on body weight are presented in Figure 1a. Initially, SHRs had lower body weight compared to Wistar rats (factor “genotype” F(1,74) = 168.95, *p* = 0.0000). ANOVA revealed a significant “isolation” × “start-end” interaction (F(1,74) = 18.59, *p* = 0.00005) because during the experiment, body weight only increased in the SHR-S group. We did not find any other differences in the body weight.

The systolic arterial pressure values were higher in SHRs compared to Wistar rats, in accordance with their genotype (Figure 1b). RM-ANOVA showed a significant effect of the “genotype” factor (F(1,74) = 249.85, *p* = 0.0000). The effects of the “start-end” factor (F(1,74) = 67.25, *p* = 0.0000) and “genotype” × “start-end” interaction (F(1,74) = 4.66, *p* = 0.034) were also significant. However, we did not find any effect for the “isolation” factor.

The heart rate values were significantly different in rats of two genotypes studied (factor “genotype” F(1,74) = 208.65, *p* = 0.0000). The heart rate was higher in SHRs compared to Wistar rats, which supports the fact that in SHRs, sympathetic influence on heart function is stronger than in Wistar rats (Figure 1c). Repeated measurement of the heart rate after 12 weeks of social isolation did not reveal any substantial changes in this index in Wistar males, while in SHRs, it increased significantly (“start-end” factor F(1,74) = 33.11, *p* = 0.0000 and “genotype” × “start-end” interaction F(1,74) = 88.41, *p* = 0.0000). This elevated heart rate was observed in both groups of social and isolated rats (SHR-S and SHR-I, respectively), indicating that this effect was associated with the non-invasive arterial pressure measurement procedure rather than with social isolation.

### 2.2. Effect of Social Isolation on the Blood CORT Level in Rats

**Figure 2:**
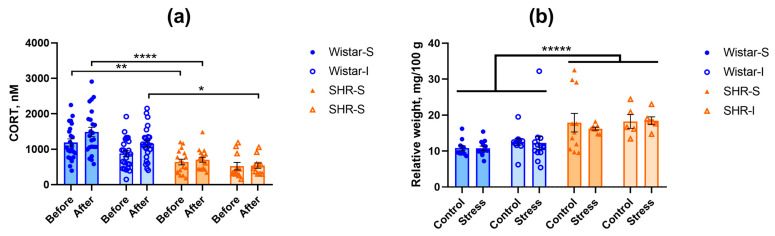
Effects of social isolation on (**a**) the CORT level in blood of Wistar rats and SHRs before and after 12 weeks of isolation (points Before and After on abscissa axis, respectively). Ordinate axis, CORT concentration, nM. The differences between the groups were calculated using repeated-measures ANOVA followed by Tukey test for unequal N for multiple comparisons. The differences are significant at *, *p* < 0.05, **, *p* < 0.01, and ****, *p* < 0.0001 according to the post hoc Tuley test for unequal N. (**b**) Relative adrenal weight in isolated control groups (Control) and groups subjected to acute restraint stress (Stress). Ordinate axis: relative weight of the adrenal glands, mg/100 g of body weight. The differences between two genotypes are significant at *****, *p* < 0.00001 according to the Mann–Whitney U-test. Wistar-S and SHR-S are the groups of rats housed in group conditions, and Wistar-I and SHR-I are the groups of rats housed in isolated conditions. Data are presented as M ± s.e.m.

In order to estimate the stress effect of social isolation, we measured the level of CORT in peripheral blood 12 weeks after the start of isolation. The CORT concentration was significantly lower in SHRs compared to Wistar rats (Figure 2a). ANOVA revealed the effects of “genotype” (F(1,72) = 40.71, *p* = 0.0000) and “isolation” (F(1,72) = 6.42, *p* = 0.0135) without interaction between these factors (F(1,72) = 0.95, *p* = 0.3321). The level of CORT increased to some extent 12 weeks after the start of experimental treatment (factor “start-end” F(1,72) = 8.20, *p* = 0.0055) with significant “genotype” × “start-end” interaction (F(1,72) = 4.59, *p* = 0.0355). This was associated with a very subtle elevation in CORT concentration in Wistar rats; however, this effect was observed in both groups of socially housed and isolated rats (Wistar-S and Wistar-I, respectively), again showing no effect of social isolation on the level of CORT. Interestingly, the lower CORT concentration in SHRs compared to Wistar rats was related to adrenal hypertrophy in SHRs (U = 79, Z = −6.19, *p* = 0.0000, according to the Mann–Whitney U-test). Data on the relative adrenal weight are presented in Figure 2b. Thus, animals of the two genotypes studied significantly differed in concentrations of circulating CORT, which was probably related to adrenal hypertrophy in SHRs. Twelve-week housing of Wistar rats under experimental conditions subtly increased the CORT concentration, but this effect did not depend on social isolation, and it could not be found in the post hoc comparison.

### 2.3. Differential Effect of Social Isolation on the Changes in Glucose and CORT Concentrations During Acute Restraint Stress

**Figure 3:**
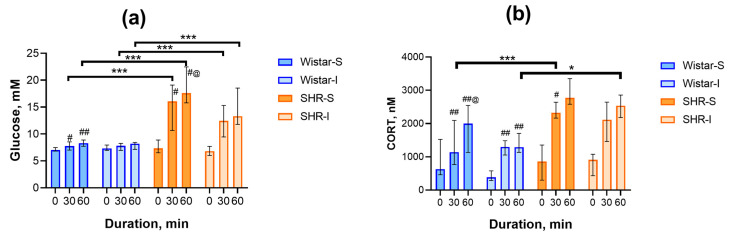
Time course of changes in glucose and CORT concentrations in rat blood during acute restraint stress. (**a**) Glucose concentrations in the blood sampled 0, 30, or 60 min after the start of restraint. Ordinate axis: glucose concentration, mM; abcissa axis: duration of restraining, min. The differences are significant at #, *p* < 0.05 and ##, *p* < 0.01, vs. point 0 and @, *p* < 0.05 vs. point 30 min according to the Wilcoxon test corrected for multiple comparisons; ***, *p* < 0.001 according to the post hoc z’-test. (**b**) CORT concentrations in the blood plasma sampled 0, 30, or 60 min after the start of restraint. Ordinate axis: CORT concentration, nM; abcissa axis, duration of restraining, min. Wistar-S and SHR-S are the groups of rats housed in group conditions, and Wistar-I and SHR-I are the groups of rats housed in isolated conditions. The differences are significant at #, *p* < 0.05 and ##, *p* < 0.01, vs. point 0 and @, *p* < 0.05 vs. point 30 min according to the Wilcoxon test corrected for multiple comparisons; *, *p* < 0.05 and ***, *p* < 0.001 according to the post hoc z’-test. Data are presented as median (LQ-UQ).

To answer the question of whether isolation can modify the body’s response to acute heterotypic stress, we studied the glucose and CORT concentrations in peripheral blood during 1 h of restraint. Immediately after placing the rats into the restrainers, the levels of glucose did not differ in the Wistar-S and Wistar-I groups (7.1 ± 0.1 and 7.4 ± 0.2 mM, respectively) and in the SHR-S and SHR-I groups (7.6 ± 0.4 и 7.0 ± 0.3 mM, respectively). Repeated-measures Friedman ANOVA revealed a significant increase in the glucose concentration in the Wistar-S group (Figure 3a; (χ^2^(N = 12, df = 2) = 12.13, *p* = 0.0023), whereas in the Wistar-I group, this growth was practically not observed (χ^2^(N = 13, df = 2) = 0.76, *p* = 0.68). In the SHR-S and SHR-I groups, the glucose concentration increased significantly during restraint (Figure 3a; χ^2^(N = 8, df = 2) = 8.00, *p* = 0.0047 and χ^2^(N = 6, df = 2) = 12.0, *p* = 0.0025, respectively). However, post hoc corrected multiple comparison of mean ranks revealed a significant increase between the time points 0–30, 0–60, and 30–60 min in the SHR-S group only. In the SHR-I group, the level of significance was very close but not less than the *p*-value (0.0277 vs. 0.017) corrected for comparison of the three groups. The elevation in glucose concentration was more expressed in SHRs compared to Wistar rats at 30 min (H(3, N = 39) = 27.25, *p* = 0.0000) and 60 min (H(3, N = 39) = 29.04, *p* = 0.0000) after the start of restraint, independently of the preliminary housing conditions. Taking into account that alterations in the glucose level during stress may reflect SAS reactivity to some extent, we can assume that SAS reactivity was more expressed in hypertensive compared to normotensive animals. Moreover, long-term isolation of normotensive rats could either decrease SAS responsiveness to acute heterotypic stress or reflect the exhaustion of this system due to isolation.

As expected, acute restraint stress triggered the secretion of CORT into the blood plasma. The statistically significant increase in the CORT concentration was observed in both groups of normotensive animals (χ^2^(N = 12, df = 2) = 22.17, *p* = 0.00002 and χ^2^(N = 13, df = 2) = 19.85, *p* = 0.00005 for Wistar-S and Wistar-I, respectively; Figure 3b); however, the time course of this elevation was different in these groups. In Wistar-S, CORT levels exhibited a continuous increase, culminating in a maximal value by the end of the restraint period. Conversely, Wistar-I rats displayed a blunted response with a modest rise in CORT content during the initial 30 min, followed by a lack of further increase. In the SHR-S and SHR-I groups (Figure 3b), CORT levels also significantly increased (χ^2^(N = 8, df = 2) = 9.75, *p* = 0.0076 and χ^2^(N = 6, df = 2) = 9.33, *p* = 0.0094, respectively) with a rise up to maximal values during 30 min after the start of restraint stress. Similarly to glucose alterations, the differences between specific time points in the SHR-I were insignificant when taking into account the corrected *p*-value.

CORT concentrations were similar in the groups studied immediately after the start of restraint (H(3, N = 39) = 5.69, *p* = 0.13). Thirty minutes after the start of the restraint period, the differences between the rats of the two genotypes became clearly visible (H(3, N = 39) = 18.21, *p* = 0.0004). The rise in CORT was 90% higher and appeared earlier in the SHR-S group compared to the Wistar-S group. Though a similar rise by 80% was revealed in the SHR-I group, this effect was statistically insignificant (*p* > 0.11). The differences between the groups were also observed after 60 min of restraint (H(3, N = 39) = 14.58, *p* = 0.0022); however, the alterations were different. Thus, in the Wistar-S group, the CORT concentration increased additionally, in contrast to the SHR-S group, which led to the disappearance of a significant difference between these groups. The blunted CORT response in the Wistar-I group and an increase in the SHR-I group led to the appearance of a statistically significant difference between these groups at this time point. Thus, long-term social isolation in rats of two genotypes studied affected the function of the HPA system and its capability to respond to acute restraint stress.

### 2.4. Social Isolation and Acute Restraint Did Not Affect the Levels of mRNA of Glucocorticoid Signaling-Associated Genes in the Hippocampus and Frontal Cortex of Wistar and SHR Rats

**Figure 4:**
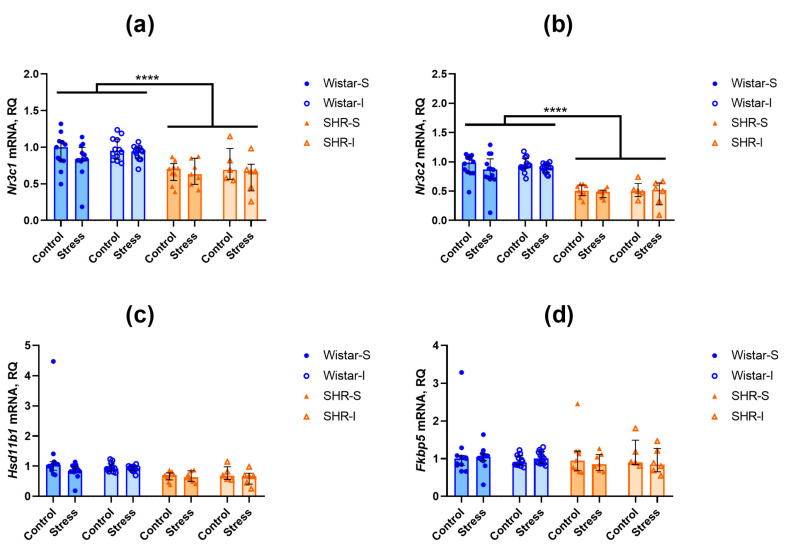
Effects of social isolation and acute restraint on the mRNA levels in the frontal cortex of Wistar rats and SHRs. Wistar-S and SHR-S are the groups of rats housed in group conditions, and Wistar-I and SHR-I are the groups of rats housed in isolated conditions. Control and Stress are subgroups of each group not subjected or subjected to 1 h restraint. The contents of transcripts: (**a**) *Nr3c1* (glucocorticoid receptor); (**b**) *Nr3c2* (mineralocorticoid receptor); (**c**) *Hsd11b1* (11β-hydroxysteroid dehydrogenase type 1); (**d**) *Fkbp5* (FK-506-binding protein 51 (FKBP5/FKBP51)). Ordinate axis: transcript content and relative quantity (RQ). The differences are significant at ****, *p* < 0.0001 according to the Mann–Whitney U-test. Data are presented as median (LQ-UQ).

**Figure 5:**
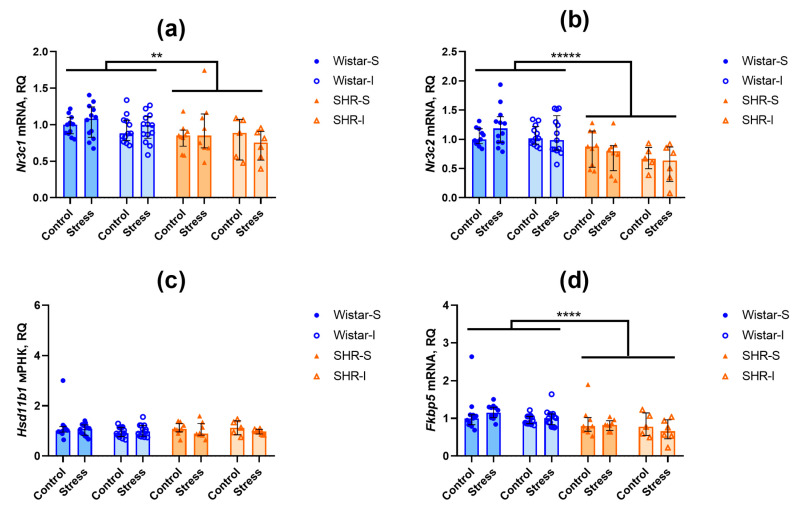
Effects of social isolation and acute restraint on the mRNA levels in the hippocampus of Wistar rats and SHRs. Wistar-S and SHR-S are the groups of rats housed in group conditions, and Wistar-I and SHR-I are the groups of rats housed in isolated conditions. Control and Stress are subgroups of each group not subjected or subjected to 1 h restraint. The contents of transcripts: (**a**) *Nr3c1* (glucocorticoid receptor); (**b**) *Nr3c2* (mineralocorticoid receptor); (**c**) *Hsd11b1* (11β-hydroxysteroid dehydrogenase type 1); (**d**) *Fkbp5* (FK-506-binding protein 51 (FKBP5/FKBP51)). Ordinate axis: transcript content and relative quantity (RQ). The differences are significant at **, *p* < 0.01, ****, *p* < 0.0001, and *****, *p* < 0.00001 according to the Mann–Whitney U-test. Data are presented as median (LQ-UQ).

The contents of *Nr3c1* transcripts related to glucocorticoid receptor were lower in the hippocampus (U = 235, Z = 4.55, *p* = 0.000005) and frontal cortex (U = 204, Z = 4.75, *p* = 0.000002) of SHRs compared to Wistar rats (Figure 4a and Figure 5a). A similar difference was revealed in the levels of *Nr3c2* mRNA of the gene encoding the mineralocorticoid receptor in the frontal cortex (Figure 4b; U = 45, Z = 6.55, *p* = 0.00000) and hippocampus (Figure 5b; U = 425, Z = 2.46, *p* = 0.014).

The expression of *Hsd11b1* mRNA, encoding an activator of glucocorticoids, did not differ in both brain structures studied (Figure 4c and Figure 5c). In the hippocampus, the expression of *Fkbp5* mRNA, a negative modulator of glucocorticoid and mineralocorticoid receptors, was lower in SHRs compared to Wistar rats (Figure 5d; U = 249, Z = 4.40, *p* = 0.00001), whereas in the frontal cortex (Figure 4d), the level of *Fkbp5* mRNAs was similar in the rats of these two genotypes.

Thus, we can assume that prolonged housing under the isolated conditions did not substantially affect the expression of the investigated genes. Similarly, acute restraint stress failed to induce any notable alterations in the contents of the studied mRNA transcripts within the brain structures.

### 2.5. Prolonged Social Isolation Modifies the Relationships Between mRNA Expression in Hippocampus and Frontal Cortex of Wistar Rats and SHRs

**Figure 6:**
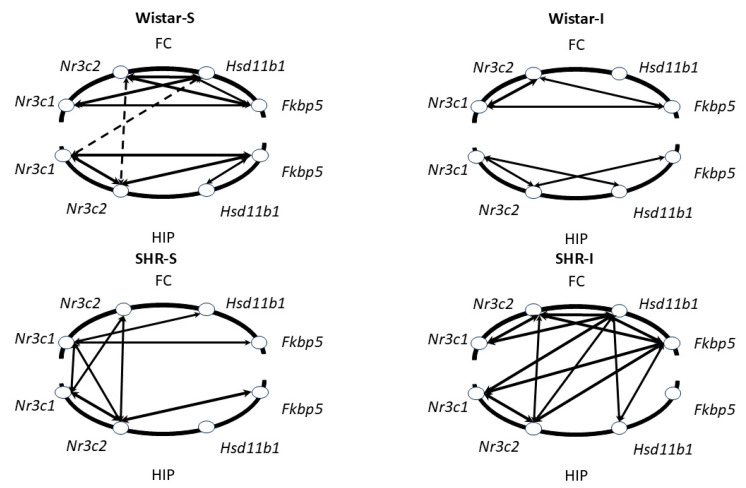
Patterns of correlations between the levels of GC signaling-associated genes’ mRNAs in the frontal cortex (FC) and hippocampus (HIP). Arrows indicate significant correlations between the specific genes within and between the brain structures. Solid and dotted arrows represent positive and negative correlations, respectively. Thickness of arrows is proportional to the values of moderate (0.5 < r_S_ < 0.7) and strong (0.7 < r_S_ < 1.0) correlations classified according to the Spearman correlation coefficients (r_S_), with significance level *p* < 0.05.

Then, we studied how hypertension, social isolation, and acute stress modified the relationships between the transcriptional activity in the brain regions studied. We calculated Spearman correlation coefficients and their changes under different experimental conditions. Taking into account that acute restraint did not influence the expression of the target genes, we combined the subgroups in each genotype to calculate correlation coefficients. We have to note that the blood CORT level did not correlate with any of the mRNA contents in the hippocampus and frontal cortex (Figure S1). However, patterns of correlations between the mRNA levels of glucocorticoid signaling-associated genes differed in specific experimental groups. In the frontal cortex of Wistar-S rats, strong and moderate correlations were revealed between the levels of all mRNA transcripts (Figure 6 and Figure S1), but in the hippocampus of these animals, a strong correlation was observed only between *Nr3c1* and *Nr3c2* mRNAs and moderate correlations between *Fkbp5* mRNA and mRNAs of three other genes. In hypertensive rats of the SHR-S group, there was another poorer pattern of significant moderate correlations for *Nr3c1* mRNA and *Fkbp5* and *Hsd11b1* mRNAs in the frontal cortex and for *Nr3c1* mRNA and *Nr3c2* mRNA, as well as *Nr3c2* mRNA and *Fkbp5* mRNA (Figure 6 and Figure S1). Some moderate correlations in transcriptional activity were revealed between the brain structures. Thus, in the Wistar-S group, almost all correlations between the hippocampus and frontal cortex were negative, although only two of them were significant (Figure 6 and Figure S1), whereas in the SHR-S group, all the correlations were positive. These differences in the orchestration of transcriptional activity in the brains of normotensive and hypertensive rats may indicate substantial modification of glucocorticoid-associated signaling under chronic hypertensive conditions.

Prolonged social isolation modified correlation patterns in rats of each genotype. Thus, in the frontal cortex of the Wistar-I group, only correlations between *Nr3c1* and *Nr3c2* mRNA, as well as *Fkbp5* mRNA and *Nr3c1* and *Nr3c2* mRNAs, were revealed (Figure 6 and Figure S1). Moreover, the coefficient of *Fkbp5* mRNA and *Nr3c1* mRNA correlation decreased significantly (*p* < 0.01) in the Wistar-I group compared to the Wistar-S group. In the hippocampus of the Wistar-S group, the number of correlations also decreased, and the coefficient values for remaining correlations between *Nr3c1* and *Nr3c2* mRNAs and *Fkbp5* and *Nr3c1* mRNAs were significantly lower (*p* < 0.01) than those found in Wistar-S rats. Correlations between the frontal cortex and hippocampus were not observed.

Prolonged social isolation in hypertensive animals led to the appearance of a net of strong correlations in the frontal cortex, unlike the SHR-S group (Figure 6 and Figure S1). The only correlation coefficient found in the hippocampus of the SHR-I group between *Nr3c1* and *Nr3c2* mRNAs increased significantly (*p* < 0.01) in comparison to that observed in the SHR-S group, indicating possible strengthening of a connection between the transcription activity of the genes encoding glucocorticoid and mineralocorticoid receptors. The number of correlations between the frontal cortex and the hippocampus also increased in the SHR-I compared to the SHR-S group. Thus, the patterns of correlations between the mRNAs of glucocorticoid signaling-associated genes in the brains of normotensive and hypertensive animals differed; prolonged social isolation caused a significant restructuring of the correlation patterns, a “mismatch” of mRNA expression in normotensive Wistar rats, and greater “consistency” in hypertensive SHRs.

## 3. Discussion

Taken together, our results show that prolonged housing of rats in individual cages is associated with complex and sometimes oppositely directed changes in the physiological, biochemical, and molecular indicators of the stress-responsive systems of the body in the animals with normal (Wistar) or chronically elevated (SHR) arterial pressure. The main finding is that 14-week social isolation, per se, subtly affected baseline indices of the functional state of the SAS and HPA systems, leading to a stress response and the expression of glucocorticoid signaling-associated genes in the animals’ brains. However, some intimate but important modulating effects of chronic social isolation were revealed, which were differently manifested in the animals of the two genotypes studied.

### 3.1. Genotype-Related Differences as a Basis for Manifestation of the Effects of Social Isolation

Our results largely support most data reported in the literature on the differences between normotensive Wistar rats and chronically hypertensive SHRs. Although the exact cause of essential hypertension in SHRs is not known, among possible factors, along with vascular changes, endothelial dysfunction, inflammation, and oxidative stress, increased activity of the renin–angiotensin–aldosterone system and the sympathetic nervous system are often observed. Even under resting conditions, SHRs exhibit higher epinephrine, but not norepinephrine or dopamine excretion [43]. They also have higher basal activity of the SAS [14,15,44,45], and disruption of sympathetic nerves or adrenalectomy leads to a decrease in arterial pressure in rats of the SHR strain [44,46]. Lower basal concentration of circulating CORT associated with adrenal hypertrophy may reflect reduced reactivity of the HPA system or its adaptation to chronic sympathetic activation [47]. Decreased expression of glucocorticoid and mineralocorticoid receptors encoding genes in the frontal cortex and hippocampus in SHRs compared to Wistar rats may indicate impaired negative feedback and resistance to glucocorticoids in this animal model of hypertension. However, data on the expression of glucocorticoid receptors in the frontal cortex and hippocampus of SHRs are controversial. Some authors reported no difference in the glucocorticoid and mineralocorticoid receptors in the hippocampus of SHRs and normotensive WKY rats [48], whereas others found increased mineralocorticoid receptor expression in the hippocampus of SHRs and considered this fact a reason for the development of encephalopathy [49].

In contrast to SHRs, Wistar rats have a more balanced stress-responsive system. Therefore, they demonstrated a response closer to the expected and “normal” [3] response to stressors. It seems that these genotypic differences are central to understanding the other effects of social isolation, because they initially affected different “targets”.

### 3.2. Social Isolation Has Limited Effects on Basal Physiological Indicators and mRNA Expression in the Studied Brain Structures

Monotonous life, hypokinesia due to the small size of cages, limited sensory information, and social contact were the main factors influencing animals during individual housing. In the present study, we did not reveal any substantial effect of social isolation on the studied physiological indicators. At least partially, this was probably due to the age of the animals used in the experiment. After one year, they did not exhibit extensive body weight gain. However, in the SHR-S group, we observed a small increase in body weight, while isolation prevented an increase in body weight in the SHR-I group, probably because of the stressogenic effect of individual housing. Decreased systolic arterial pressure was observed in all the groups independently of the genotype or housing conditions. This was probably due to the fact that 10–13-month-old animals were used in the present study, which distinguishes our experiment from that performed earlier, in which juvenile SHRs were isolated [39]. Subtle effects of social isolation on basal physiological parameters were also reported by other authors who used adult rats for the experiments [50]; however, this did not prevent the development of cognitive deficit, emotional disturbance, and numerous molecular alterations in the brain of isolated animals. On the other hand, prolonged isolation of adult rats may be related to less expressed or more compensated changes in the monoamine system in the hypothalamus in comparison to short-term isolation [33]. Thus, the absence of robust physiological alterations does not mean the absence of the effects of individual housing.

An increase in heart rate observed in SHRs but not Wistar rats was probably induced by the repeated-measurement procedure, which was conducted in restrainers, i.e., increased responsiveness to acute stress.

We revealed a very small increase in basal CORT concentration using ANOVA, but this was not followed by significant differences between the groups of control and isolated animals in both genotypes. In the literature, it is widely discussed that loneliness in people or social isolation in animals does not always lead to a simple and linear increase in the basal indices, such as cortisol or CORT, but rather evoke more complex modifications, including changes in their circadian rhythms, rate of negative feedback, and, most importantly, hyperresponsiveness of the system to acute challenges [33,34,51,52]. Notably, in our experiment, isolation did not result in significant changes in any of the studied genes associated with glucocorticoid signaling, such as *Nr3c1*, *Nr3c2*, *Fkbp5*, and *Hsd11b1*, in the frontal cortex or hippocampus. This may indicate the resilience of the transcription apparatus to the stressogenic effect of social isolation. These data are in line with previous studies demonstrating that the long-term effects of stress are typically not due to alterations in basal expression levels, but rather to epigenetic changes that modify the capacity of gene expression in response to future challenges [53,54]. Thus, in adult mice isolated for 3 months, increased activity of DNA-methyltransferases, histone deacetylases, histone methyltransferases, and histone acetyltransferases was revealed in the midbrain [55], indirectly supporting the idea of a wide spectrum of epigenetic alterations during chronic isolation and their contribution to the modification of the phenotype of isolated animals in previous and present studies.

### 3.3. Social Isolation Modulates the Body’s Response to Acute Stress

The effects of social isolation were more apparent after exposure to acute restraint stress. Thus, the glucose and CORT responses were blunted during restraint in the Wistar-I group, which probably reflected an attenuation of sympathetic tone or SAS desensitization and HPA system exhaustion developed during 14-week isolation. These effects may be a result of the increased allostatic load [2,56]. In SHRs, the changes in glucose and CORT concentrations developed more rapidly, peaking after 30 min of the restrained period. In SHRs, isolation did not significantly affect indicators of stress-responsiveness in blood, probably due to high sympathetic tone or adrenal hypertrophy, which are both specific features of this genotype. Interestingly, the changes in stress-responsiveness after chronic exposure to different stressors vary significantly. Thus, overcrowding of rats, a psychosocial stressor, led to desensitization of the HPA system, whereas cold exposure for 1 or 8 weeks sensitized the HPA system to the action of a heterotypic stressor, such as ether inspiration [57]. Housing rats in the colony of subordinants, a psychosocial stressor, for 3 weeks enhanced the HPA response to the action of an acute heterotypic stressor despite the fact that, under in vitro conditions, the reaction of the adrenal glands to adrenocorticotropin was decreased [58]. In humans, loneliness also differently affected stress-responsiveness. Analysis of eleven studies in a review [59] showed that in some experiments, there was sensitization, but in others, there was desensitization of the reaction to acute stress among persons with a high sense of loneliness. Neither sex nor the nature of stressors contributed significantly to this reaction, but loneliness substantially influenced the capacities of the cardiovascular system, increasing arterial pressure and stimulating inflammation. Our data show no changes in arterial pressure after social isolation in rats. We can assume that all these data demonstrate that the mechanism of regulation of stress-responsiveness cannot be explained by the functioning of direct connections or feedback within the HPA system. It seems that this mechanism involves more complex regulatory systems in the brain.

### 3.4. Social Isolation Differently Modifies the Patterns of Correlations Between the Studied Indices in the Brains of Wistar Rats and SHRs

It has been shown that the analysis of correlations among some biochemical indices may be used as a fruitful approach to the analysis of interactions between brain structures in acute stress [60] or stress-associated learning [61]. Here, we studied the patterns of correlations between the levels of mRNA transcripts of genes related to glucocorticoid signaling in the frontal cortex and hippocampus. In the frontal cortex of the Wistar-S group, there were correlations between all mRNA transcripts, except for Fkbp5 mRNA, and vice versa. In the hippocampus, there were only correlations between the level of Fkbp5 mRNA with other transcripts and mRNA contents of the genes encoding glucocorticoid and mineralocorticoid receptors. Correlations between the frontal cortex and hippocampus, structures involved in the central control of negative feedback within the HPA system [3,6,62], were less expressed. In the SHR-S group, dysfunction of the HPA system was associated with less expressed relationships in the contents of the investigated transcripts in the frontal cortex and hippocampus in comparison with the Wistar-S group. In contrast to Wistar-S rats, who had subtle negative correlations between the frontal cortex and hippocampus, all interstructural correlations in the SHR-S group were positive. Prolonged social isolation changed both the r_S_-values and the correlation patterns, and these changes were oppositely directed in rats of two genotypes studied. In the Wistar-I group, this was expressed as a “mismatch” between the correlation net and a decrease in r_S_-values in each of the structures and between them. Hypothetically, this may reflect impairment of functional connections between brain structures caused by isolation-associated stress. It is well known that chronic restraint stress leads to shortening of apical dendrites with a decrease in the spine density in pyramidal neurons of the frontal cortex [63]. In rats, this restructuring of projection neurons in the frontal cortex after chronic stress can impair brain functions such as working memory, attention switching, or cognitive flexibility [64]. It was previously shown that Wistar rats exhibited cognitive impairments after chronic isolation [65]. In the SHR-I group, we revealed an increase in the “consistency” of transcriptional machinery inside the frontal cortex and hippocampus and between them. This was specifically expressed for *Hsd11b1* and *Fkbp5* mRNA. This may indicate some rapid and consistent increase in the transcriptional activity of genes contributing not so much to an increase in potential capability of brain tissue to receive the glucocorticoid signal via increased number of receptors, but rather to changes in the signaling mechanisms at the level of regulatory proteins responsible for the activation of glucocorticoids, such as Hsd11b1, or the modulation of glucocorticoid receptors and mineralocorticoid receptors, such as FKBP5. Increased “consistency” of transcription processes across different brain structures of SHR-I rats indicates greater coordination of molecular networks and/or increased rigidity of the system or decreased plasticity, which can be observed in neuronal networks during the development of pathological conditions [66].

## 4. Materials and Methods

### 4.1. Animals

**Figure 7:**
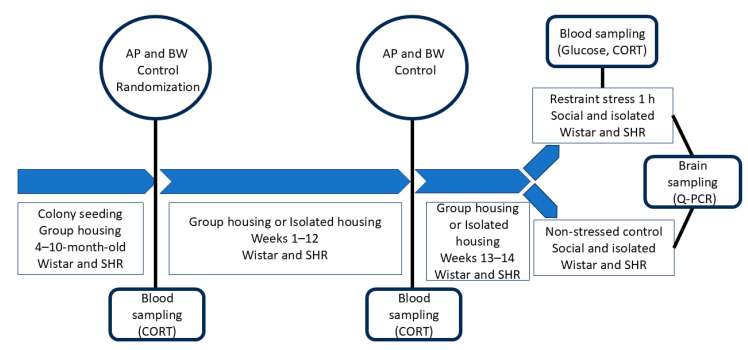
A schematic drawing of the experimental timetable. AP, arterial pressure; BW, body weight; CORT, corticosterone; Q-PCR, real-time polymerase chain reaction. Four-month-old Wistar and SHR rats were used. They were maintained in groups before the age of 10 months. After AP and BW control and blood sampling, they were distributed into 4 experimental groups: isolated rats and group-housed rats of each strain. The period of isolation continued for 12 weeks, and AP, BW, and CORT were controlled. After additional 2 weeks, the animals of each group were divided into 2 subgroups, one of which was subjected to 1 h of restraint stress. After this procedure, all animals were euthanized.

The experiment was conducted on 50 male Wistar rats and 34 male SHR rats. The rats were supplied by the Animal Breeding Facility (the Unique Research Unit Bio-Model of the Shemyakin and Ovchinnikov Institute of Bioorganic Chemistry, Russian Academy of Sciences, Moscow Region, Russia) at the age of 4 months. A total of 3–4 rats were housed per cage, which was made of clear plastic, in the institutional animal facility room under 12 h light/dark conditions (light on 8.00 a.m.) and access to fresh water and food ad libitum. The experiment was started at the age of 10 months. A timetable of the experimental protocol is presented in Figure 7.

### 4.2. Social Isolation

At the start of the experiment, half of the animals of each genotype (25 Wistar rats and 16 SHRs) were individually placed into tight opaque white plastic cages (200 × 120 × 120 mm in size), whereas the other half (25 Wistar rats and 18 SHRs) was left in home cages (480 × 375 × 210 mm in size) in groups of 2–3 animals. The animals were housed under these conditions for 15 weeks. Individual cages and home cages were located in one room near opposite walls; therefore, the rats could communicate with each other via odor or ultrasound signals. The litter was changed in all cages daily. For this purpose, the rats were gently transferred with a gloved hand, litter was removed, the cage was rapidly washed with water and filled with fresh litter, and the rats were returned to their home cages. This procedure took no more than 5 min. Water and food remained available ad libitum during the whole experiment. The rats were weighed before the start and after the end of the experiment. Finally, four groups of rats were formed: Wistar-S, Wistar-I, SHR-S, and SHR-I. The mortality rate was as follows: in Wistar-S, *n* = 3; Wistar-I, *n* = 1; SHR-S, *n* = 2; SHR-I, *n* = 6 (the differences between the groups, Wistar-I and SHR-I, were significant according to the Fisher exact test, *p* = 0.026).

### 4.3. Measurement of Arterial Pressure and Heart Rate

Arterial pressure was measured in rats using the tail cuff method. The rats were preliminarily adapted to plastic restrainers thrice (once a day). All measurements were performed between 14.00 and 17.00 h, i.e., during the light phase of a day cycle. After this, they were placed into restrainers again and warmed using a “Flogiston” heating pad (Neurobotics, Zelenograd, Moscow, Russia), and a stable temperature was maintained during the procedure. A cuff was put onto the tail and connected to a “Systola” device (Neurobotics, Zelenograd, Moscow, Russia). Arterial pressure and heart rate were recorded using “Systola” software (v. 2.0.0.) provided by the manufacturer.

### 4.4. Acute Restraint Stress

Acute restraint stress was modeled by placing the rat into a tight Plexiglas restrainer for 1 h. For this purpose, the rats of the groups Wistar-S, *n* = 12; Wistar-I, *n* = 13; SHR-S, *n* = 8; SHR-I, *n* = 6 were pseudorandomly selected according to their body weight. Restraint stress was initiated 3 h after the light was switched on. The animals were transported in a separate room for this procedure in their home cages. All procedures were conducted within 2 h and were applied at the same time for all animals used. After the end of the procedure, the rats were anesthetized with 3% isoflurane and decapitated. Control animals were also transported in a separate room in their home cages, anesthetized, and decapitated in parallel to the stressed rats.

### 4.5. Collection of Biological Samples

For CORT analysis, blood was sampled in heparinized tubes via the tail vein puncture method both before and after 12 weeks of isolation, with measurements taken 3 h after the start of the light phase of a day cycle. The sampling was performed under short-time isoflurane anesthesia. For assessment of stress responsiveness, CORT was also assayed in blood samples 0, 30, and 60 min after the start of acute stress. For glucose measurement, a fresh blood drop was sampled 0, 30, and 60 min after the start of acute stress. The tail was cleared with a cotton pad wetted with water and 70% ethanol; the vein was visualized and punctured. The blood (200 µL) was collected and then centrifuged at 1500× *g* for 15 min, and plasma samples were collected.

After decapitation, the brain was taken out of the skull and washed in ice-cold isotonic saline solution, and the frontal cortex and hippocampus were dissected on ice and weighed. All the samples were frozen in liquid nitrogen and stored at −86 °C until analysis.

### 4.6. CORT and Glucose Measurement

Glucose was measured in the blood drop using a One-Touch Select Plus glucometer (LifeScan Europe GmbH, Zug, Switzerland) and respective test strips.

CORT content was measured using the “Corticosterone, Rat” kit (HEMA, Moscow, Russia) for enzyme-linked immunosorbent assay according to the protocol of the manufacturer.

### 4.7. RNA Extraction and Quantitative Polymerase Chain Reaction

Total RNA was extracted from the brain samples using Extract RNA reagent (#BC032, Evrogen, Moscow, Russia) according to the manufacturer’s protocol. A part of RNA was treated with DNAse I (Thermo Fisher Scientific, Waltham, MA USA), and the other part was used as a negative control without reverse transcription. Synthesis of cDNA was performed using an equimolar mixture of random decaprimer and oligo(dT)-primer (#SB002 and #SB001, Evrogen, Moscow, Russia), MMLV RT Kit (#SK021, Evrogen, Russia), and the RNA inhibitor RiboCare (#EK005M, Evrogen, Moscow, Russia) according to the manufacturer’s protocol. The product of reverse transcription was diluted at a ratio of 1:7.

The level of mRNA transcripts was measured with a qPCRmix-HS SYBR + LowROX ready-to-use mixture (#PK156L, Evrogen, Moscow, Russia) using a CFX384 DNA amplificator (Bio-Rad, Hercules, CA USA). The levels of mRNA *Hsd11b1*, *Fkbp5*, *Nr3c1*, *Nr3c2*, *Ywhaz*, and *Hprt1* genes were estimated. Primer sequences were chosen using the NCBI database and Lasergene Primer Select Software Package (release 18, DNASTAR, Madison, WI, USA). All the sequences are presented in Table 1. The relative quantity of transcripts was calculated using the 2^−ΔΔCT^ method, taking into account the reaction efficacy relative to the expression of *Ywhaz* and *Hprt1* mRNAs, and the data are presented as relative units (RQ).

### 4.8. Data Analysis

The correspondence of data in samples to the normal distribution was estimated using the Shapiro–Wilk test. Differences between the groups in body weight, systolic arterial pressure, heart rate, and CORT level after 12-week social isolation were estimated using the analysis of variances for repeated measures (RM-ANOVA) with the Tukey post hoc test for unequal N. Between-factors were “genotype” and “isolation”, and within-factor was “start-end”. Changes in the contents of glucose and CORT in blood plasma during acute stress were analyzed using non-parametric tests because they did not pass the Levene test for variance homogeneity. Thus, we used the Kruskal–Wallis test followed by multiple rank comparison according to the z’-test by Siegel–Castellan for independent variables and Friedman ANOVA for ranks, followed by the Wilcoxon test corrected for multiple comparisons. Differences were considered significant at *p* < 0.017. The expression of genes in different groups and conditions was estimated using the Mann–Whitney U-test corrected for multiple comparisons. In this case, the differences were considered significant at *p* < 0.01. For analysis of relationships between the indices studied and brain structures, Spearman correlation coefficients (r_S_) were calculated. Only statistically significant correlations were considered with *p* < 0.05, and correlations were considered as strong (0.7 < r_S_ < 1) or moderate (0.5 < r_S_ < 0.7). The values of Spearman correlation coefficients were compared with each other after Fisher’s z-transformation.

## 5. Conclusions

We showed that prolonged housing of rats evoked complex, sometimes opposite, changes in the physiological, biochemical, and molecular indices of the body’s stress-responsive systems in normotensive and hypertensive animals. Regulation of the stress response seems to be more complex and not limited only by the feedback system within the hypothalamus–pituitary–adrenocortical or sympatho-adrenomedullary systems, but operates at the level of the limbic system and the cerebral cortex. Further, an arrangement of the transcriptional machinery of the glucocorticoid signaling-associated genes differs in the normotensive and hypertensive animals of the studied genotypes, and social isolation stress led to oppositely directed changes in the patterns of correlations in their expression within and between the hippocampus and frontal cortex. Thus, we can hypothesize that the genotype associated with chronic hypertension in SHRs itself causes persistent alterations in the brain. These changes affect the expression of the genes involved in glucocorticoid signaling and the brain’s response to social stress. This response was more evident in the organization of transcriptional processes across the studied brain regions rather than in direct alterations of transcript levels.

## 6. Limitations of the Study

There are several limitations that should be taken into account for the correct interpretation of the data presented in this study. Firstly, in the present study, the animals were subjected to daily care with litter change due to increased urination in SHRs. Thus, this daily handling could affect the stress-associated biochemical indicators measured in this study. Therefore, we could not determine the effect of isolation on CORT concentration in blood plasma. However, in the previous sections, we presented data from multiple other studies demonstrating the absence of CORT changes reported by different groups using various subjects and species. In our study, the daily change of litter was applied to both isolated and social groups of both genotypes. Therefore, we can assume that the absence of clear changes in CORT concentration was probably due to the adaptation of animals to the housing conditions rather than a lack of effect of isolation. Secondly, the concentration of circulating CORT depends on many variables, including circadian rhythm, housing conditions, animal care, experimental procedures, and many others. Taking this into account, we performed all the experimental procedures at the same time of day in order to prevent the effect of circadian fluctuations. The animals were transported from the housing facilities to the experimental room as soon as possible in their home cages; the interval between the transfer and the time of restraint stress exposure or euthanasia was also shortened. CORT levels in this study seem to be relatively high. As we mentioned, the level of CORT depends on many factors and specific experimental procedures, such as the type of blood collection, the type of assay, and others. In the present study, we used blood collected from the tail vein under anesthesia to estimate CORT before and after 12 weeks of isolation and from non-anesthetized rats during exposure to acute stress. This may explain some differences observed between the isolation-related CORT level and its level at point 0 of the restraint stress procedure. On the other hand, this allowed us to observe a clear physiological response to acute stress in Wistar-S, SHR-S, and SHR-I groups of rats. Further, the blunted response of CORT in the Wistar-I group may be caused by isolation housing, because a similar blunted response was observed for glucose in the same group. Thirdly, we did not find any changes in the levels of mRNA transcripts of the genes associated with glucocorticoid signaling in brain structures after acute restraint stress. This was probably due to the inappropriate timing for brain sample collection chosen in this study. Changes in gene expression require more time, at least 2 h, to be detected. In the present study, we decapitated animals within 15 min of the end of stress exposure. However, this rapid euthanasia allowed us to demonstrate that the isolation of rats of different genotypes substantially modified hypothetical complex mechanisms of transcriptional activity in the frontal cortex and hippocampus. In future studies, the delayed effects of acute restraint stress on the expression of genes associated with glucocorticoid signaling after chronic social isolation will be investigated in more detail.

## Figures and Tables

**Table 1:** Sequences of primers for Q-PCR.

Gene	Forward Primer	Reverse Primer	E
*Hprt* NM_012583.2	CGT CGT GAT TAG TGA TGA TGA AC	CAA GTC TTT CAG TCC TGT CCA TA	2
*Ywhaz* NM_013011.4	TTG AGC AGA AGA CGG AAG GT	GAA GCA TTG GGG ATC AAG AA	2
*Nr3c1* NM_001408897.1	TCC CCC TGG TAG AGA CGA AGT	CTC CCC TGG CCA AGC AAA CTG	1.93
*Nr3c2* NM_013131.2	GAC CTT GGA GCG TTC TTC AC	AAT CTC CAT GTA GTT GTT CTC AGT G	2
*Hsd11b1* NM_017080.2	GCC TGG GAG GTT GTA GAA AGA G	AAT AGT AGT AAC CCA GGC AGA GCA C	1.93
*Fkbp5* NM_134455.2	GCC GGC AAG AAA CAC GAG AGT	GAG GAG GGC CGA GTT CATT AGGA	2

## Data Availability

The original contributions presented in this study are included in the article/Supplementary Materials. Further inquiries can be directed to the corresponding author.

## Supplementary Materials

The supporting information can be downloaded at: https://www.mdpi.com/article/10.3390/ijms262412050/s1.

## Abbreviations

CORT	Corticosterone
HPA system	Hypothalamic–pituitary–adrenocortical system
SAS	Sympatho-adrenomedullary system
SHR	Spontaneously hypertensive rat
SHR-I	SHR rats housed in isolated conditions
SHR-S	SHR rats housed in group conditions
Wistar-I	Wistar rats housed in isolated conditions
Wistar-S	Wistar rats housed in group conditions
