# Supplementary material for: Glucocorticoid Signaling-Associated Gene Expression in the Hippocampus and Frontal Cortex of Chronically Isolated Normotensive and Hypertensive Rats and the Responsiveness to Acute Restraint Stress
Source: Int J Mol Sci. 2025 Dec 15;26(24):12050. doi: 10.3390/ijms262412050 (PMC12732442; doi:10.3390/ijms262412050)
Supplement: Supplementary file 1 [file ijms-26-12050-s001.zip › ijms-4003437-supplementary.pdf]

Wistar-S								
	FC				HIP			
	Hsd11b1	*Nr3c1*	*Nr3c2*	*Fkbp5*	*Hsd11b1*	*Nr3c1*	*Nr3c2*	*Fkbp5*
Hsd11b1	1.000							
Nr3c1	0.760	1.000						
Nr3c2	0.863	0.813	1.000					
Fkbp5	0.542	0.779	0.549	1.000				
Hsd11b1	0.047	0.196	0.001	0.288	1.000			
Nr3c1	-0.372	-0.215	-0.365	0.113	0.296	1.000		
Nr3c2	-0.442	-0.309	-0.535	-0.026	0.304	0.906	1.000	
Fkbp5	-0.320	-0.027	-0.346	0.408	0.479	0.751	0.701	1.000

Wistar-I								
	FC				HIP			
	Hsd11b1	*Nr3c1*	*Nr3c2*	*Fkbp5*	*Hsd11b1*	*Nr3c1*	*Nr3c2*	*Fkbp5*
Hsd11b1	1.000							
Nr3c1	0.172	1.000						
Nr3c2	0.028	0.823	1.000					
Fkbp5	0.205	0.471	0.645	1.000				
Hsd11b1	0.256	-0.163	-0.171	-0.145	1.000			
Nr3c1	0.079	-0.305	-0.262	-0.176	0.508	1.000		
Nr3c2	-0.265	-0.196	-0.122	-0.161	0.235	0.662	1.000	
Fkbp5	-0.032	-0.320	-0.219	0.343	0.375	0.337	0.419	1.000

SHR-S								
	FC				HIP			
	Hsd11b1	*Nr3c1*	*Nr3c2*	*Fkbp5*	*Hsd11b1*	*Nr3c1*	*Nr3c2*	*Fkbp5*
Hsd11b1	1.000							
Nr3c1	0.603	1.000						
Nr3c2	0.138	0.418	1.000					
Fkbp5	0.456	0.556	0.259	1.000				
Hsd11b1	0.400	-0.032	0.382	0.039	1.000			
Nr3c1	0.189	0.543	0.636	0.164	0.414	1.000		
Nr3c2	0.114	0.546	0.536	0.325	0.054	0.700	1.000	
Fkbp5	0.279	0.164	0.282	0.468	0.507	0.475	0.568	1.000

SHR-I								
	FC				HIP			
	Hsd11b1	*Nr3c1*	*Nr3c2*	*Fkbp5*	*Hsd11b1*	*Nr3c1*	*Nr3c2*	*Fkbp5*
Hsd11b1	1.000							
Nr3c1	0.818	1.000						
Nr3c2	0.791	0.900	1.000					
Fkbp5	0.791	0.555	0.664	1.000				
Hsd11b1	0.682	0.509	0.545	0.673	1.000			
Nr3c1	0.755	0.509	0.564	0.782	0.418	1.000		
Nr3c2	0.664	0.527	0.645	0.745	0.482	0.909	1.000	
Fkbp5	0.327	0.000	-0.064	0.100	0.327	0.509	0.364	1.000

Figure S1. Correlation matrices for different animal groups used in the study. Spearman correlation coefficients of the contents of genes encoding proteins involved in corticosterone signaling mRNAs in the frontal cortex (FC) and hippocampus (HIP) are presented. Values of r_S varied from -1 (green) to +1 (red). Statistically significant correlation(r_S values with $p < 0.05$) are indicated in red. Isolated groups are Wistar-I (n = 24) and SHR-I (n = 14); social groups are Wistar-S (n = 22) and SHR-S (n = 13).
